# The development of depression and social anxiety symptoms in adolescents and the negative impact of the COVID-19 pandemic and desire for peer contact

**DOI:** 10.3389/fpubh.2024.1374327

**Published:** 2024-09-17

**Authors:** Anne L. Pinkse-Schepers, J. Marieke Buil, Hester Sijtsma, Miriam Hollarek, Reubs J. Walsh, Mariët van Buuren, Lydia Krabbendam, Nikki C. Lee

**Affiliations:** ^1^Section of Clinical Developmental Psychology, Vrije Universiteit Amsterdam, Amsterdam, Netherlands; ^2^Levvel, Amsterdam, Netherlands; ^3^Einstein Lab of Cognitive Neuroscience, Gender and Health, University of Toronto, Toronto, ON, Canada; ^4^Department of Developmental Psychology, Utrecht University, Utrecht, Netherlands

**Keywords:** adolescents, depression, social anxiety, peer contact, social distancing, COVID-19

## Abstract

**Introduction:**

Adolescence is a dynamic developmental phase in which contact with peers is crucial for socio-emotional development and wellbeing. Depression and social anxiety show patterns of high onset during this period, and more for girls than boys. Here we examine this development among Dutch adolescents, as well as how desire for more peer contact as a result of social distancing measures during the COVID-19 pandemic contributed to this increase.

**Methods:**

We used a longitudinal three-wave design to examine 406 typically developing Dutch adolescents across two consecutive cohorts; Cohort 1: 2016–2019 (*N* = 138, 53.6% girls, age at T0 *M* = 13.00, *SD* = 0.42), Cohort 2: 2017–2020 (*N* = 268, 63.1% girls, age at T0 *M* = 13.05, *SD* = 0.39), final wave during spring 2020 during the first COVID-19 lockdown. Self-report questionnaires were used to measure depression and social anxiety symptoms, desire for change in the amount of peer contact during lockdowns, and emotion regulation. Parallel process dual latent growth models and autoregressive cross-lagged models were used to test the hypotheses.

**Results:**

Results showed that symptoms of both depression and social anxiety increase during adolescence. Gender analysis reveal a higher initial level and increase in depression symptoms for girls, while levels for boys decreased. Adolescents exposed to the pandemic showed a steeper increase in depression but not in social anxiety. Desire for more peer contact was related to an increase in depression and social anxiety, though only in girls. No evidence was found for moderation of emotion regulation skills concerning COVID evoked emotions on the association between desire for peer contact and anxiety and depression symptom development.

**Discussion:**

Symptoms of social anxiety increased during adolescence in boys and girls. Symptoms of depression increased for girls, but decreased for boys. The increase in depression was greater in a cohort who experienced the COVID-19 pandemic. For girls, a desire for more peer contact was associated with an increase of depression and social anxiety symptoms in times of social restrictions.

## 1 Introduction

Adolescence is a dynamic developmental stage, defined by remarkable changes at the physical, psychological and social level (1). Interactions with friends become of vital importance to achieve the developmental tasks of this period (2). Furthermore, adolescence is characterized by an increase in different forms of psychopathology. The rise in internalizing symptoms (such as social anxiety and depression) is especially pronounced, with a higher risk and more severe course in girls compared to boys (3, 4). There is evidence that the increase in psychopathology was exacerbated during the COVID-19 pandemic (5). An import factor contributing to the impact of COVID-19 on adolescent mental health may have been the social distancing measures, which in the Netherlands meant that schools were closed and adolescents had very limited face-to face interactions with people outside their family. This decreased adolescents’ contact with peers during a developmental period characterized by an increased importance of peer interactions (6). This study aims to investigate the extent to which desire for more peer contact during the lockdown impacted mental health in this sensitive developmental stage. We compared the development of symptoms of depression and social anxiety in a cohort of adolescents (average 13–15 years of age) who experienced the pandemic and subsequent social distancing measures and a cohort who did not, and considered the association between desire for more peer contact and internalizing psychopathology.

Typical adolescent development is characterized by a heightened risk of internalizing symptoms, particularly of emotional problems such as symptoms of depression and social anxiety (7, 8). Depression is twice as prevalent in adolescent girls compared to boys. This difference becomes apparent after the start of puberty (9), with adolescent girls generally experiencing higher mean levels of depressive symptoms compared to boys from approximately age 13 onwards (10). Symptoms of social anxiety are high during early adolescence and seem to increase during adolescence (11). There is some evidence for a slight decrease in social anxiety symptoms during mid-adolescence and another rise in late-adolescence (12), but other studies show a stable pattern (13, 14). Prevalence is higher in girls (3), with 1.7 girls for every 1 boy diagnosed with social anxiety disorder (7). Some studies have reported a decrease in social anxiety in boys between the ages of 11 and 15 (15, 16).

The social environment is thought to play a significant role in the increase in symptoms of depression and social anxiety during adolescence. As the importance of being part of the peer group rises, so do the consequences of negative social experiences such as rejection and exclusion (17). The development of depressive symptoms is bidirectionally associated with poorer quality and less closeness in peer relationships (18, 19). On the other hand, close peer relationships and perceived friendship support may have a protective effect on resilient functioning (20). Social isolation, reduced social contact and loneliness during adolescence is also associated to higher risks of depression and social anxiety (21–24). Contrarily, having more (intimacy in) friendships protected adolescents against feelings of social anxiety (18). Girls experience a greater impact of the social environment on the development of depression or social anxiety, possibly related to higher social needs, higher social sensitivity and skills and higher demands on these social skills placed on girls in many cultures (25, 26). Given this social reorientation during adolescence, and strong focus on peers, the social restrictions during the COVID-19 pandemic may have had a large impact on adolescent mental health and specifically internalizing psychopathology.

Many adolescents have reported increased levels of depression and anxiety, among other mental health problems, during and after the COVID-19 pandemic. A meta-analysis including 43 studies among adolescents across multiple continents (Europe, the USA and Asia) showed that studies reported elevated levels of group as well as within-person depression symptoms during the pandemic, and a greater increase than expected in levels of depression symptoms (5). This effect was observed in girls, while the evidence in boys was inconclusive. Less evidence is available for social anxiety. Two cross-sectional studies reported that self- or parent-reported social anxiety showed (near) significant increases during the pandemic in Turkey (27) and China (28), and three longitudinal studies found significant increases in social anxiety at various timepoints; pre- to post-pandemic among early adolescents in the USA (29), after the first peak of COVID-19 among late adolescents in the USA (30) and in Finland after 1 year of COVID-19 (31). The latter reported steeper increases for girls than for boys, while the other studies found no gender differences (30) or did not report them.

Governmental restrictions were associated with changes in depression and general anxiety symptoms. A meta-analysis of longitudinal studies concluded that in areas with stronger governmental restrictions depression symptoms increased more and general anxiety symptoms decreased less in adolescents pre- to during COVID-19 (32). A few studies focussed on social impact of the distancing measures. In studies of more general indicators of mental health, adolescents explicitly pointed out that the factor causing them the most COVID-related stress was not seeing their friends (33). More girls than boys reported that this was negatively impacting their mental health (57.8% vs. 26.3%) (34). Feeling socially connected during the pandemic protected against development of depression and anxiety (33). One study considered actual peer contact and found that high social anxiety was associated with both increases and with decreases in contact with friends (31). This unexpected effect in both directions might reflect the complexity of social contact during the pandemic. In the absence of day-to-day contact with peers (e.g., at school) some adolescents may have become anxious and sought reassurance through increased contact with peers (e.g., via social media), while others may have withdrawn.

Taken together, there is evidence for elevated levels of symptoms of depression in adolescents during COVID-19, with higher increases for girls than for boys. There are fewer studies on social anxiety, but these point in the same direction. There is some evidence that these effects were associated with reduced peer contact. An important transdiagnostic factor in the onset and maintenance of depression and social anxiety are emotion regulation skills (35, 36). Emotion regulation strategies are used by people to maintain, decrease, or increase emotions (37). The use of strategies such as reflection, reappraisal, distraction and social sharing are generally considered as adaptive, whereas expressive suppression and rumination may be maladaptive (38). Emotion regulation skills can play an important role in whether stressful life events lead to the development of depression or anxiety or not, especially in adolescence (39). In general, the use of maladaptive emotion regulation strategies is positively correlated with symptoms of depression and anxiety during adolescence, while the opposite is true for the use of adaptive strategies (35). In adults, vulnerability for developing depression or anxiety during the pandemic correlated with maladaptive emotion regulation (40). The current study investigated the development of symptoms of depression and social anxiety during adolescence, as well as the impact of reductions in peer contact due to social distancing measures and the possible moderating role of emotion regulation for COVID-19 provoked emotions. We followed two cohorts (the first from 2016 to 2019, the second from 2017 to 2020) of Dutch adolescents during the first 3 years of secondary school using an observational, longitudinal design, with four aims. The first aim was to study the developmental pathway of symptoms of depression and social anxiety from early to mid-adolescence in our first cohort (consisting of adolescents tested prior to the COVID-19 pandemic) as this is a key period of risk for the emergence of these symptoms. We expected an increase with age for both symptoms of depression and social anxiety, and more in girls than in boys. Secondly, we investigated the impact of exposure to COVID-19 pandemic on these developmental pathways by comparing the pathways of cohort one and two. We expected a stronger increase in symptoms of depression and social anxiety in cohort two, the cohort that was exposed to the pandemic, again more in girls than in boys. The third aim was to investigate whether desire for social contact with peers during the first lockdown was associated with the development of symptoms of depression and social anxiety in the second cohort. During this lockdown period in the Netherlands, adolescents were unable to: go to school or other activities outside the home, have others visit their home, meet more than two people at a time outside. They were also required to keep a physical distance to each other of 1.5 m from people outside their household. These regulations dramatically decreased their opportunity to interact face-to-face with peers. Therefore, we expected that a desire for more social contact with peers was associated with a rise in both symptoms of depression and social anxiety during COVID-19, and more so in girls than in boys. Finally, we examined a possible moderation effect of emotion regulation in response to emotions provoked by situations resulting from the COVID pandemic on the association between desire for social contact with peers and changes of symptoms of depression and social anxiety during COVID-19. We hypothesized that adolescents who used more adaptive emotion regulation skills during the pandemic, show less impact of desire for social contact on the development of depression and social anxiety as they were more able to cope with these changes.

## 2 Materials and methods

### 2.1 Participants

The study was part of the longitudinal #So Connect research project [see, e.g., (41)]. In this project, data was collected about symptoms of depression and social anxiety from 840 adolescents across two cohorts (51.8% girls; age at start *M* = 13.03, *SD* = 0.42). They were living in the Netherlands and were enrolled in mainstream secondary schools, situated in both rural and urban areas in the Netherlands. Adolescents were recruited during their first year of secondary school and, over the course of the project, adolescents participated in six waves of data collection (two per school year, one in the autumn and one in the spring with six-month intervals, in which only the waves in the spring included the questionnaires about psychopathology used in the current study). For the current study, data from the waves assessing psychopathology were included in the analyses (wave two, hereafter referred to as T0; wave four, hereafter referred to as T1 and wave six, hereafter referred to as T2). Data were collected in two consecutive cohorts: cohort 1 (2016–2019, 138 adolescents (T0) from two secondary schools, age T0: *M* = 13.00, *SD* = 0.42, range 11.70–13.73, age at T2: *M* = 14.98 *SD* = 0.39 range 13.76–14.98, 53.6% girls) and cohort 2 (2017–2020, 268 adolescents (at T0) from the same two schools as cohort 1 as well as five additional schools, age T0: *M* = 13.05, *SD* = 0.39, range 11.67–14.06, age at T2 *M* = 15.08 *SD* = 0.41 range 13.40–15.08, 63.1% girls). The two cohorts were enrolled in one of the two upper secondary school tracks of the Dutch education system (top ∼40% of pupils based on academic achievement). In a subset of the sample, we used postal codes to measure social economic background characteristics. This showed that the majority of the pupils came from families at or above the median income. The two cohorts were comparable in terms of location of the schools, level of education and country of birth. Schools were selected on basis of their locations that cover highly urban, small/suburban towns, and rural areas. The sample was highly ethnically homogeneous, with less than 1% born in not Western European countries. Cohort 1 and 2 were equivalent in base levels for depression symptoms as measured with the Center for Epidemiologic Studies Depression scale (CES-D (42); *M* = 13.76 vs. 14.69, *t* = −1.086, *p* = 0.28, Cohen’s d = −0.089, df = 771, 95% CI [−2.6, 0.75]) or social anxiety as measured with the Social Anxiety Scale for Adolescents (SAS-A (43); *M* = 38.48 vs. 38.89, *t* = −0.407, *p* = 0.899, Cohen’s d = 0.034, df = 762, 95% CI [−2.4, 1.6]). For cohort 2, the final measurement took place during spring 2020 when the first COVID-19 lockdown was implemented. All participants and their parents provided informed consent. The study protocol was approved by the Institutional Ethics Board of the Vrije Universiteit Amsterdam [Vaste Commissie Wetenschap en Ethiek (VCWE)].

As class composition changed between school years, additional adolescents joined participating classes and subsequently were invited to join the study. This led to inclusion of new participants in the later waves of the study. Based on the complete sample 773 participated in the second wave of data collection (T0 in the current analyses), 651 in the fourth (T1) and 452 in the sixth wave (T2). All adolescents with complete data at T1 and T2 were included in the study, while missing data at T0 was allowed, to minimize the effect of selective attrition due to the COVID-19 pandemic. Drop out was higher for cohort 2, T2 (50.3%) than for other timepoints (cohort 2, T0; 6.4%, T1; 19.2% and cohort 1, T0; 13.7%, T1; 25.6% and T2; 33.9%) as data collection was conducted online due to the COVID-19 lockdown and subsequent school closures. Comparison of the subsample used in the current study (*n* = 406, 59.6% girls; age at T0 *M* = 13.03, *SD* = 0.40) to those that were excluded showed that the excluded group had significantly higher scores for depression symptoms at T0 as measured with the CES-D; *M* = 15.44 vs. 13.41, *t* = 2.718, *p =* 0.007, Cohen’s d *=* 0.196, df *=* 771, but not at T1; *M* = 15.18 vs. 14.47, *t* = 0.780, *p* = 0.436, Cohen’s d = −0.063, df = 649. For social anxiety as measured with the SAS-A there were no differences at T0; *M* = 39.24 vs. 38.31, *t* = 1.072, *p = 0*.284, Cohen’s d *=* 0.078, df *=* 762, nor at T1; *M* = 40.36 vs. 40.89, *t* = 0.534, *p* = 0.600, Cohen’s d = 0.042, df = 649. The subsample also did not differ in age (0.00 year older) but consisted of more girls (8.1% more) than the total sample (*n* = 840). Comparison of the included sample of cohort 2 to those that were excluded of C2 due to dropout at T2, showed a similar pattern: depression symptoms at T0 as measured with the CES-D; *M* = 15.84 vs. 13.52, *t* = 2.641, *p =* 0.008, Cohen’s d *=* 0.22, df *=* 572, but not at T1; *M* = 14.63 vs. 15.16, *t* = −0.510, *p* = 0.610, Cohen’s d = 0.04, df = 480.

### 2.2 Design

**Figure 1:**
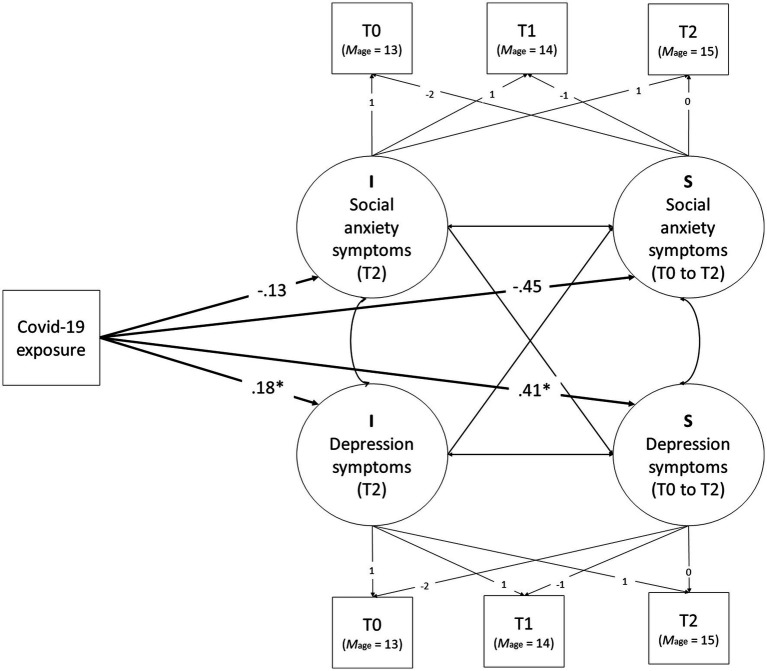
Graphical representation of the Measurement and Structural Model of the effects of COVID-19 exposure on the levels and development of symptoms of depression and social anxiety.

The study has a longitudinal, quasi-experimental design, using one cohort to study the developmental pathway of depression and social anxiety symptoms, and one cohort to evaluate the impact of COVID-19 and subsequent desire for more peer contact on the developmental pathway of depression and social anxiety (see Figure 1). Data for all timepoints were collected in the classroom environment, except T2 of cohort two, which was conducted online due to school closures during the COVID-19 lockdown. This timepoint was conducted in June 2020, over the course of 2 weeks, in the last week of 12 weeks of school closure for most participants and for a few in the first week of the reopening of schools. Questions about the pandemic explicitly referred to the quarantine period.

### 2.3 Measures and procedure

#### 2.3.1 Depression

Symptoms of depression were measured using a 20-item, Dutch version of the CES-D for children [CES-DC (44, 45)]. The questionnaire includes items regarding psychological, cognitive and somatic symptoms of depression, providing a well-rounded measure of depressive symptoms with good validity and stability for screening in adolescents (46). On a 4-point Likert scale (0, seldom to 3, most of the time or always), adolescents indicated the frequency with which they experienced a symptom during the last week. In the present study, internal consistency reliability (Cronbach’s alpha) was good with 0.91 (T0), 0.91 (T1), and 0.94 (T2). Sum scores of level of depression symptoms were used in the analyses. Scores range from 0 to 60, with higher scores indicating more symptoms of depression.

#### 2.3.2 Social anxiety

Symptoms of social anxiety were measured using the Social Anxiety Scale for Adolescents [SAS-A (43, 47); translated by Blöte]. The SAS-A is a 22-item self-report questionnaire designed to measure adolescents’ subjective experience of social anxiety. It contains 18 items related to anxiety in social situations with peers and four filler items. Each item was rated on a 5-point Likert scale ranging from 1 (“Not at all”) to 5 (“All the time”). The internal consistency of the SAS-A in the present study was good with α = 0.89 (T0), 0.90 (T1) and 0.90 (T2). Sum scores of social anxiety were used in the analyses. Scores range from 18 to 90, with higher scores indicating more symptoms of social anxiety.

#### 2.3.3 Desire for peer contact

Adolescents in cohort 2 completed additional questionnaires during T2 about their experiences with the pandemic. Desire for contact with peers was measured asking participants to what extent they would like to change the social contact they had with their friends during this period, answered on a 5-point Likert scale ranging from 1 (“a lot less contact”) to 5 (“a lot more contact”).

#### 2.3.4 Emotion regulation strategies during the pandemic

In order to measure adolescents’ use of emotion regulation strategies in response to emotions provoked by the COVID-19 pandemic, we adapted questions developed by Brans and colleagues (38) to measure the use of six strategies: reflection, reappraisal, distraction, social sharing, expressive suppression and rumination. Questions were reformulated to apply to the COVID-19 period and context (see supporting information). Each strategy was measured using a single item such as “To what extent do you search for distractions to avoid having to think about the corona virus?” (distraction). Items were rated on a 5-point Likert scale from 1 (not at all) to 5 (very much so). In previous studies, reflection, reappraisal, distraction and social sharing have been associated with increases in positive affect and decreases in negative affect, while rumination and expressive suppression have been associated with increases in negative affect and decreases in positive affect (38, 48, 49). We calculated a scale for adaptive and maladaptive strategies by summing the responses to the corresponding questions. The responses for rumination and expressive suppression reflected maladaptive strategies and the responses for reflection, reappraisal, distraction and social sharing reflected adaptive strategies. Cronbach’s alfa’s were acceptable (0.777 for the maladaptive scale and 0.700 for the adaptive scale).

#### 2.3.5 Gender

Gender was reported by the participant during every wave choosing from the question: I am: boy, girl, other, namely… It was coded as 0 = boy, 1 = girl. There were no participants who chose the answer “other.”

#### 2.3.6 Age

Age was measured via self-report of participants’ birthdate and calculated using the test date.

### 2.4 Statistical analyses

Parallel process, dual latent growth models (LGMs) were used to address the first research question on the development of symptoms of depression and social anxiety in adolescents who were not exposed to the COVID-19 pandemic from T0 to T2. In the LGMs, the development of symptoms of depression and social anxiety was estimated using continuous latent growth factors. The latent intercept (or mean) represents the expected score on T0 and the latent slope represents linear change in levels of depression symptoms and social anxiety over time (i.e., from T0 to T2). Multiple group comparisons were used to test for potential gender differences in the level of depression and social anxiety symptoms at T0 and the developmental patterns in internalizing psychopathology over time. Departing from a model that assumed no gender differences, model fit of nested models in which the parameters of interest were freed sequentially was compared using the (strictly positive) Satorra Bentler chi-square difference test (50, 51).

Our second aim to investigate main effects of the COVID-19 period on the level of and change in depression symptoms and social anxiety was tested by regressing the latent intercept and latent slope on COVID-19 exposure (yes/no; intercept centered at T2; see Figure 1 for a graphical presentation of this model). The latent intercepts were centered at T2 to test for level differences in symptoms of depression and social anxiety between the two groups after the COVID-19 group was exposed, but before the non-COVID group was exposed. Furthermore, we tested for level differences between the exposed and non-exposed group in the latent intercepts of social anxiety and depression symptoms in the pre-COVID-19 period as well (i.e., at T0 and T1) to test for level differences in outcomes not due to COVID-19 exposure (i.e., potential cohort effects). In addition, the main effect of COVID-19 on the level and developmental change of social anxiety and depression symptoms was tested for moderation by gender.

The effect of the desire for peer contact on the relative change in depressive symptoms and social anxiety from T1 to T2 for the second cohort (i.e., the cohort exposed to COVID-19)- our third research aim - was tested in an autoregressive cross-lagged path model. In addition, the association between the desire for social contact with peers and the relative change in symptoms of depression and social anxiety from T1 to T2 was tested for possible moderation effects by gender. Lastly, our fourth and final research aim was addressed by testing whether association between desire for peer contact and the relative change in depressive symptoms and social anxiety from T1 to T2 was moderated by adaptive and maladaptive emotional regulation strategies.

Models were fitted in Mplus 8.10 (52). Models were estimated using the robust maximum likelihood estimator (MLR) to account for non-normal distribution of the study variables. Standard errors of the estimates were adjusted to account for clustering of participants within schools using a sandwich estimator (53). Missing data were handled using Full Information Maximum Likelihood (FIML) estimation. All estimates, except for the unconditional models, were controlled for gender and age differences when appropriate. Model fit was based on the chi-square test, the Comparative Fit Index (CFI) with values >0.90 indicating acceptable fit and values >0.95 indicating close fit, the Standardized Root Mean Square Residual (SRMR), with a value of ≤0.08 indicating acceptable fit; and the Root Mean Square Error of Approximation (RMSEA), with values ≤0.06 indicating acceptable fit [see (54, 55)]. See Table 1 for the report model fit according to CFI, RMSEA and SRMR for the baseline model (M0; intercepts and slopes fixed to be equal) and our final model. Mplus code and output files (including missing data patterns), and ΔSB-chi square calculations are available in the Open Science Framework (OSF), via the following link: https://osf.io/uews2/?view_only=d84056b928954348a76e0240ae533494

**Table 1:** Model fit indices according to CFI, RMSEA, and SRMR for the baseline model (M0; intercepts and slopes fixed to be equal) and our final model.

	CFI	RMSEA	SRMR
Baseline model	0.938	0.145	0.116
Final model	0.974	0.100	0.070
Change in fit	Δ+0.036	Δ−0.045	Δ−0.046
Conclusion	Final model fits best		

## 3 Results

### 3.1 Descriptive statistics

Means and variances of the latent intercepts and slopes of depression symptoms and social anxiety for the exposed and non-exposed group separately are in Table 2. Descriptive for the COVID-19 related questions are in Table 3. Very few adolescents desired less contact with peers, whereas the majority of adolescents were either content with the amount of contact they had with peers (40.5%) or desired more peer contact (52.3%).

**Table 2:** Means and variances of intercepts and the slope for symptoms of depression and social anxiety.

	Latent intercepts									Latent slope		
	T0			T1			T2					
	*M*	*SD*	VAR	*M*	*SD*	VAR	*M*	*SD*	VAR	*M*	*SD*	VAR
Exposed to COVID-19 (*n* = 268)												
Symptoms of depression	13.29*	7.49	56.05*	15.18*	8.37	70.07*	17.07*	4.12	117.00*	1.89*	4.06	16.45*
Social anxiety	39.31*	10.02	100.45*	40.17*	9.47	89.76*	41.35*	11.10	123.15*	1.02	4.69	22.04*
**Not exposed to COVID-19 (*n* = 138)**												
Symptoms of depression	13.32*	7.41	54.87*	14.01*	7.95	63.15*	14.70*	9.71	94.35*	0.69*	3.38	11.46
Social anxiety	37.93*	11.02	121.53*	40.33*	10.14	102.75*	42.45*	10.07	101.42*	2.26*	2.95	8.73

Means of intercepts are adjusted for age and gender differences; variances are not adjusted for age and gender differences. M = Mean; SD = Standard Deviation; VAR = Variance. *p < 0.05. Fit unconditional multiple-group model: χ^2^ (8) = 33.978, *p* < 0.001; CFI = 0.977; SRMR = 0.032; RMSEA = 0.126 [90% CI = 0.085 (lower limit), 0.172 (upper limit)]; fit model controlled for age and gender effects: χ^2^ (25) = 51.324, *p* = 0.002; CFI = 0.979; SRMR = 0.051; RMSEA = 0.072 [90% CI = 0.043 (lower limit), 0.100 (upper limit)].

**Table 3:** Percentages of desire for contact with peers.

	Desire for contact with peers				
	A lot less	Less	Same	More	A lot more
Total	3.4% *N* = 9	3.8% *N* = 10	40.5% *N* = 107	36% *N* = 95	16.3% *N* = 43
Girls	1.2% *N* = 2	4.8% *N* = 8	40.4% *N* = 57	35.5% *N* = 59	18.1% *N* = 30
Boys	7.1% *N* = 7	2.0% *N* = 2	40.8% *N* = 40	36.7% *N* = 36	13.3% *N* = 13

### 3.2 Development of internalizing psychopathology and gender difference analyses

The unconditional parallel-process LGM for the non-COVID group only showed adequate fit to the data (χ^2^ = 5.460, df = 4, *p* = 0.243; CFI = 0.994; SRMR = 0.024; RMSEA = 0.051 [90% CI = 0.000–0.147]). Both depression and social anxiety symptoms increased with age (*M* age at T0 13.00 at T1 13.96 and T2 14.98) (depressive symptoms: *M* at T0 = 13.413; slope T0-T2: *B* = 0.551, *p* < 0.001; social anxiety symptoms: *M* at T0 = 38.102, slope T0-T2: *B* = 2.073, *p* < 0.001). Furthermore, no gender differences were found in the mean at T0 and the slope of social anxiety symptoms (ΔSB chi-square difference tests, both *p* ≥ 0.273). However, girls had higher levels of depression symptoms at T0 compared to boys (*M*_girls_ = 14.442, *SE* = 0.965, *p* < 0.001; *M*_boys_ = 12.028, *SE* = 0.508, *p* < 0.001; ΔSB chi-square difference test = 176.930, df = 1, *p* < 0.001). In addition, while girls showed an increase in depression symptoms from T0 to T2, depression levels for boys decreased (*M*_girls_ = 0.913, *SE* = 0.309, *p* = 0.003; *M*_boys_ = −0.370, *SE =* 0.130, *p* = 0.004; ΔSB chi-square difference test = 4.956, df = 1, *p* = 0.026).

### 3.3 Effect of exposure to the COVID-19 period on the development of internalizing psychopathology

Fit indices showed that the parallel-process LGM in which the growth parameters were regressed on COVID-19 exposure, had adequate fit to the data (χ^2^ = 29.050, df = 14, *p* = 0.010; CFI = 0.987; SRMR = 0.041; RMSEA = 0.051 [90% CI = 0.024–0.078]). Results for depressive symptoms indicated that adolescents exposed to the COVID-19 period showed higher levels of depressive symptoms after exposure (i.e., at T2) compared to adolescents who were not exposed (effect of COVID-19 exposure: *B* = 1.806, *SE* = 0.606, *p* = 0.003, 95% CI of *B* = 0.619–2.993, β = 0.180, Cohen’s *d* = 0.172). No level differences in the intercept of depression were found pre-COVID exposure (T0: *p* = 0.628, Cohen’s *d* = −0.004; T1: *p* = 0.145; Cohen’s *d* = 0.109). Furthermore, exposed adolescents showed a larger increase in the development of depression symptoms from T0 to T2 (*B* = 1.095, *SE* = 0.512, *p* = 0.032, 95% CI of *B* = 0.093–2.098, β = 0.411, Cohen’s *d* = 0.243) compared to non-exposed adolescents.

For social anxiety, there were no level differences associated with COVID-19 after exposure (*p* = 0.071, Cohen’s *d* = −0.093). Analyses of intercept differences pre-COVID-19 exposure indicated that (to-be) exposed adolescents’ levels of social anxiety symptoms pre-exposure were not significantly different from non-exposed adolescents at T0 (*p* = 0.211; Cohen’s *d* = 0.141) and T1 (*p* = 0.946, Cohen’s *d* = 0.019). Furthermore, exposed and non-exposed adolescents showed no significant differences in levels of growth in social anxiety symptoms from T0 to T2 (*p* = 0.074, Cohen’s *d* = −0.157). Interaction analyses indicate that all COVID-19 exposure effects did not differ between boys and girls (all *p*s ≥ 0.371). For a graphical presentation of the main effects, see Figure 1. For estimates of levels and slope of symptoms of depression and social anxiety at T0, T1, and T2, per group, see Table 2.

### 3.4 Impact of desire for peer contact on the development of depressive and social anxiety symptoms

Main effects of desire for peer contact on the relative change in depression symptoms and symptoms of social anxiety from T1 to T2 in the COVID-19 group were estimated using a cross-lagged autoregressive model. The model for the group in total showed overall adequate fit to the data (χ^2^ = 4.648, df = 4, *p* = 0.325; CFI = 0.999; SRMR = 0.025; RMSEA = 0.025 [90% CI = 0.000–0.098]). Results are presented in Table 4. As can be seen, no main effect of desire for peer contact on the development of depressive symptoms and social anxiety was found.

**Table 4:** Results for main effects in cohort 2 of desire for peer contact on the development of depression symptoms and social anxiety and moderation by gender and emotion regulation strategies.

	Symptoms of depression development						Symptoms of social anxiety development					
	*B*	*SE*	95% CI of *B*		*p*	β	*B*	*SE*	95% CI of *B*		*p*	β
			LL	UL					LL	UL		
Control effects												
Age	0.598	1.400	−2.146	3.343	0.669	0.020	1.209	1.197	−1.138	3.556	0.313	0.038
Gender	2.808	1.231	0.396	5.220	0.022	0.241	0.608	1.888	−3.092	4.308	0.747	0.050
Pre-exposure dep	0.565	0.127	0.315	0.814	<0.001	0.558	0.051	0.104	−0.154	0.255	0.628	0.047
Pre-exposure anx	0.066	0.069	−0.069	0.201	0.336	0.072	0.567	0.028	0.512	0.622	<0.001	0.587
Main effects												
Peer contact	1.698	0.948	−0.160	3.556	0.073	0.134	1.317	0.772	−0.196	2.831	0.088	0.099
Moderation by gender												
Peer contact	−0.047	1.006	−2.019	1.924	0.962	−0.004	−0.765	1.086	−2.893	1.364	0.481	−0.057
Gender	2.949	1.277	0.446	5.452	0.021	0.254	0.776	1.976	−3.096	4.649	0.694	0.063
Peer contact × gender	3.098	0.964	1.208	4.988	0.001	0.185	3.696	1.116	1.508	5.883	0.001	0.209
Moderation by adaptive and maladaptive emotion regulation strategies												
Peer contact	1.553	0.771	0.041	3.065	0.044	0.126	1.085	0.685	−0.258	2.428	0.113	0.084
Adaptive ER	−0.078	0.668	−2.091	0.529	0.243	−0.062	−0.131	0.306	−0.732	0.469	0.668	−0.010
Maladaptive ER	2.176	0.669	0.865	3.486	0.001	0.162	2.113	0.770	0.602	3.623	0.006	0.148
Peer contact × adaptive ER	0.362	0.876	−1.356	2.079	0.680	0.026	−0.650	0.825	−2.266	0.967	0.431	−0.044
Peer contact × maladaptive ER	1.018	0.805	−0.560	2.597	0.206	0.069	1.074	0.899	−0.689	2.836	0.232	0.068

dep = symptoms of depression; anx = symptoms of social anxiety; ER = emotion regulation. Bold estimates are significant effects of interest.

Testing for gender differences showed interaction effects between gender and desire for peer contact for both depression symptom and anxiety symptom development (see Table 4 for estimates). In probing these effects for boys and girls, no association was found between desire for peer contact and depression development (*p* = 0.962) or anxiety development (*p* = 0.481) for boys. However, for girls, more desire for peer contact resulted in a relatively higher level of depression symptoms (*B* = 3.051, *SE* = 1.081, *p* = 0.005, 95% CI of *B* = 0.931–5.170, β = 0.243) and social anxiety (*B* = 2.931, *SE* = 0.808, *p* = 0.001, 95% CI of *B* = 1.347–4.515, β = 0.220), from T1 to T2 (pre-to during COVID-19 exposure). See Table 4 for estimates.

### 3.5 Moderation by emotion regulation

Tests of interaction effects between adaptive and maladaptive emotion regulation strategies and desire for peer contact indicated that there was no evidence that emotion regulation skills moderated the associations between desire for peer contact and the development of depression symptoms (adaptive *p* = 0.680; maladaptive *p* = 0.206) or social anxiety symptoms (adaptive *p = 0*.431; maladaptive *p* = 0.232). See Table 4 for estimates.

## 4 Discussion

This study examined the developmental pathway of symptoms of depression and social anxiety in typically developing adolescent girls and boys using a longitudinal design, as well as the impact of the specific context of the COVID-19 on these pathways. Results showed that, in line with our hypothesis, symptoms of social anxiety increased with age, though no gender differences were observed in this trajectory. Partially in line with our hypothesis, girls reported higher levels of symptoms of depression than boys at the first timepoint (average age 13) and showed increasing levels of symptoms with age, while boys showed a decrease in symptoms of depression with age. As we expected, when compared to non-exposed adolescents, symptoms of depression increased significantly more in the adolescents who were exposed to the first COVID-19 lockdown. However, no main effect was found between the two groups with regards to symptoms of social anxiety. Also contrary to our predictions, no evidence was found that COVID-19 exposure effects were different for boys and girls. Desire for more social contact with peers during the lockdown was associated with more depression symptoms and higher levels of social anxiety symptoms, but only in girls. Lastly, not in line with our expectations, there was no evidence that the ability to regulate COVID-related emotions during the pandemic moderate the association between peer contact during lockdown and development of symptoms of social anxiety and depression.

With regards to the developmental trajectories, the increase in symptoms of depression in girls is in line with earlier studies focussing on clinical depression (7, 56, 57). Gender effects are reported by others as well (4, 9). Complex interactions between for example biological factors such as hormonal changes, contextual factors such as different disadvantageous life stressors and socio-cultural factors are believed to underline these gender differences (26, 58, 59). Gender socialization theories propose that intensification of gender role expectations and societal demands during adolescence leads to boys and girls to behave differently. Girls might be socialized to be more affectionate, caring and expressive, possibly resulting in greater sensitivity to stress and other social behavior such as co-rumination (58, 59).

The observed steady increases in social anxiety symptoms are in line with other studies (11, 14). Others find an increase only in a certain age group. Nelemans et al. (14) report an increase between 12 and 15 years of age and a decrease thereafter, Van Oort et al. (12) report a decrease between 10 and 14 years of age and an increase between 14 and 16. Others report stability between the age of 12 and 16 (13). Note that due to our limited number of observations (i.e., three time-points) we were not able to investigate non-linear developmental patterns to test whether a linear or non-linear model would better fit our data. Another difference with previous work is the lack of measurable gender differences in the current study. However, reported gender differences in other studies are often small (12–14). Gender differences might be specific for certain symptoms of social anxiety such as fear of negative evaluation, social avoidance, distress in new situations and physiological symptoms (16, 60) possibly due to social role expectancies and socio-cultural differences (26). More research into specific developmental pathways of social anxiety is needed to understand the large individual differences in adolescent development of social anxiety symptoms.

In line with other research (32, 33, 61, 62) our results showed that adolescents in our second cohort who were exposed to the first 4 months of the pandemic and the subsequent lockdown had higher levels of depression symptoms than adolescents in the first cohort. Effect sizes in the current study were small but in line with other studies in the adult and adolescent population, suggesting that the pandemic had a significant negative impact on adolescent’s mood (30, 63). Conversely, we found no significant impact on levels of social anxiety. Others have suggested that a reduction in social interactions during the period of lockdown may actually have reduced stress for socially anxious adolescents. This is supported by studies finding a specific decrease in social anxiety in those adolescents who were less concerned about the constraints of being confined at home during the pandemic (30). The lack of exposure to social anxiety-provoking situations in the school environment (e.g., social groups or public speaking), may have temporarily reduced their social anxiety (32). Accordingly, COVID-related social restrictions allow for greater avoidance of social evaluation in individuals with pre-pandemic social anxiety, and this may mask their ongoing impairment (64). It is possible that this avoidance of social exposure could have negative consequences in the longer term, due to the lack of exposure possibilities and the positive reinforcement of the avoidance of social situations, which could possibly lead to more severe social anxiety after schools reopen (29). This hypothesis is underlined by a longitudinal study reporting an increase in social anxiety pre- to post pandemic (63).

In the current study, about half of all the participants stated they desired more peer contact, and this was associated with an increase in depression symptoms and social anxiety for girls. This is consistent with other studies correlating the perception of feeling less connected to friends to higher stress, depression and anxiety (30). The gender specific effect found in this study for the association between the desire for more peer contact and the increase in social anxiety is in line with one other study, linking perceived pandemic-related change in contact with friends to higher social anxiety, especially to perceived reduced contact for girls (31). It also corresponds to other studies reporting that girls experienced more loneliness during the lockdown than boys and that girls reported that not seeing their friends face to face during the quarantine negatively impacted their mental health in general, more so than boys did (34, 65). This may reflect a generally greater vulnerability of adolescent girls for changes and disconnection in peer relationships (66, 67), leading more easily to social anxiety in girls than in boys (15), or girls attributing increased internalizing symptoms to social distancing while boys did not make that attribution (25). Future research is needed to examine other explanations, such as whether this stronger effect for girls can be explained by social expectancies, different attributions or differences in desire for social support.

Finally, we found no evidence for a moderating role of either adaptive or maladaptive emotion regulation strategies for COVID-provoked emotions on the association between desire for more peer contact and symptoms of depression and social anxiety. Other studies during the pandemic found that general adaptive emotion regulation strategies partially negatively moderated the association between (different types of) pandemic related stress and adolescent general anxiety (68), and that emotion regulation difficulties increased the effect on adolescent depression (69). Possibly, the adolescents in the current study could attribute their desire for peer contact to external causes, namely the social distancing measures, resulting in more adaptive emotion regulation in terms of social anxiety and depression symptoms (58). Another possibility is that absence of this correlation is due to how the construct of emotion regulation was measured, namely with a specific focus on COVID-provoked emotions. Further research will have to consider what the impact of general emotion regulation skills is. The fact that we wanted to measure specifically COVID-related emotion regulation skills meant that adapted questions were used instead of the use of an existing instrument. Different types of emotion regulation strategies were combined into two constructs instead of measuring the impact of single strategies, because this was an initial exploration based on six questions. Other studies combine different questions into similar index scores, for example using the CERQ [see, e.g., (68, 70)]. The fact that questions were designed specifically for this study might have had impact on the validity of these indexes. However, reliability based on Cronbach’s alfa’s was acceptable. Further research could investigate if emotion regulation skills in general moderate the association between desire for peer contact and levels of depression and social anxiety.

This study had several strengths. First, this study was able to compare the developmental trajectories of internalizing symptoms in two cohorts, one of which was exposed to the COVID-19 measures. Second, due to the longitudinal design we were able to compare the actual developmental pathway of depression and social anxiety during the period when adolescents were exposed to the pandemic. Third, we were able to examine the impact of desire for peer contact during the lockdown on the development of symptoms of depression and social anxiety, in a developmental phase in which social contact is so important.

The current study also had several limitations. First, during the last assessment of the second cohort, attrition was relatively high compared to other waves as all questionnaires had to be administered online. The group that dropped out had significantly higher depression symptoms at T0 but not at T1. This was also the case when we compared the group that was exposed to COVID-19 and dropped out during the online assessment during the pandemic, to the included sample from this cohort at T0 and T1. This could mean that the most vulnerable group in our sample may have dropped out before the start of the pandemic. Other research has suggested that the adolescents that already suffered from internalizing psychopathology, were very vulnerable to the effects of the pandemic and subsequent measures (71). Therefore, the effects found in this study on the development of depression may be higher in the total population. Also, we wish to emphasize that the analyses were carried out under the assumption of the Missing at Random (MAR) mechanism. We presume that the missing data is systematically correlated with the observed data only (e.g., high depression scores at T0), suggesting that non-ignorable mechanisms are negligible (72, 73). Furthermore, as pointed earlier, FIML was used to handle missing data, generally known as a strong estimation method for missing value analysis (74). Second, because there were only three measurements per participant, we were only able to model a linear latent slope, and results regarding effects on the growth rate of internalizing psychopathology are likely underestimated. Furthermore, potential non-linear developmental patterns (e.g., accelerated growth after prolonged exposure) could not be tested. During the pandemic there may have been a general reluctance toward face-to-face contact within the population, which could affect the reported desire for in-person contact. However, our measure of desire for peer contact included online forms of contact, thereby circumventing measurement issues resulting from potential fears for in-person contact. Finally, we measured emotion regulation specifically in relation to the COVID-19 pandemic, making the results less comparable to studies focusing on emotion regulation in general.

Future research is needed to clarify possible long term consequences of this period of reduced peer contact for internalizing psychopathology as well as other outcomes. Studies such as the HBSC cohort (8) are important to continue to monitor the impact of the pandemic on this vulnerable age group. Another direction for future research includes how social media use influences the interplay between peer contact and mental health during pandemics, as much social contact in this period has been online (75, 76). In addition, the periods of lockdowns and school closures posed many other challenges for adolescents which may have impacted their development. These include the disruption to learning, delays in exams and the effects increased exposure to negative interactions in the home environment, such as domestic violence (77, 78).

To conclude, in a Dutch sample of typically developing adolescents there was an increase in symptoms of social anxiety for boys and girls and in depression for girls, whereas boys showed a decrease in depression symptoms, between the average age of 13 and 15. Exposure to the COVID-19 pandemic was associated with an increase in self-reported symptoms of depression and anxiety. For adolescent girls, this increase was moderated by their desire for more peer contact.

## Data Availability

The datasets presented in this article are not readily available because the participants and their parents did not provide explicit consent for public archiving of the research data, therefore the data is not stored in a public repository. Anonymized data will be made available to individual researchers upon request, when compatible with the General Data Protection Regulation. Additionally, researchers request that the data will be required to have obtained ethics approval from their host institution and are not allowed to share the data. Requests to access the datasets should be directed to a.l.pinkse-schepers@vu.nl.
