# Supplementary material for: The development of depression and social anxiety symptoms in adolescents and the negative impact of the COVID-19 pandemic and desire for peer contact
Source: Front Public Health. 2024 Sep 17;12:1374327. doi: 10.3389/fpubh.2024.1374327 (PMC11442221; doi:10.3389/fpubh.2024.1374327)
Supplement: Supplementary file 1 [file Data_Sheet_1.DOCX]

Supplementary Material: *Emotion regulation strategies used in response to emotions provoked by the COVID pandemic.*

Below are stated the questions that were used to estimate the use of emotion regulation skills for COVID-19 provoked emotions. All questions were rated by the participants from 1 (not at all) to 5 (very much so). Questions are first stated in Dutch, as they were used and then translated into English.

1. *Zoek je afleiding om niet aan het coronavirus te hoeven denken?*
   – To what extent do you search for distractions to avoid having to think about the corona virus?
2. *Onderdruk je negatieve emoties die gerelateerd zijn aan het coronavirus?*
   – Do you suppress negative emotions that are related to the corona virus?
3. *Blijf je denken aan negatieve gevoelens die veroorzaakt zijn door het coronavirus?*
   – Do you continuously think about negative emotions provoked by the corona virus?
4. *Probeer je meer positieve emoties te voelen als je je slecht voelt door het coronavirus?*
   - Do you try to feel more positive emotions when you feel bad because of the corona virus?
5. *Probeer je kalm te blijven in deze “coronasituatie”?*
   - Do you try to stay calm in this “corona situation”?
6. *Praat je met anderen over jouw gevoelens veroorzaakt door de “coronasituatie”?*
   - Do you talk with others about your feelings provoked by the “corona situation”?
